# Sedation and Gastrointestinal Adverse Event Reporting for Suzetrigine Versus Traditional Opioids: A Cohort-Restricted Disproportionality Analysis

**DOI:** 10.7759/cureus.117786

**Published:** 2026-10-11

**Authors:** Dhruv Patel, Akshita Wadhwa, Gabrielle Norman, Juliana F De Camargo, Sonia Daryanani

**Affiliations:** 1 Osteopathic Medicine, Dr. Kiran C. Patel College of Osteopathic Medicine, Nova Southeastern University, Clearwater, USA; 2 Internal Medicine, Dr. Kiran C. Patel College of Osteopathic Medicine, Nova Southeastern University, Clearwater, USA

**Keywords:** acute pain, disproportionality analysis, faers, opioids, pharmacovigilance, reporting odds ratio, suzetrigine

## Abstract

Background

Suzetrigine is a non-opioid analgesic approved for moderate-to-severe acute pain. Whether its early postmarketing adverse-event reporting differs from traditional opioids is uncertain. We tested a prespecified respiratory-depression question and secondary sedation and gastrointestinal (GI) reporting domains.

Methods

We analyzed US Food and Drug Administration Adverse Event Reporting System/Adverse Event Monitoring System quarterly American Standard Code for Information Interchange extracts from 2025 Q2 through 2026 Q1. After latest-version deduplication, the main cohort comprised adult or age-missing US reports listing suzetrigine or exactly one of oxycodone, hydromorphone, morphine, or tramadol as the primary suspect; misuse/overdose-related reports were excluded, but the cohort was not indication-matched. The primary endpoint was the exact MedDRA preferred term “Respiratory depression,” with minimum counts of 10 suzetrigine and 20 comparator reports. Secondary domains were sedation and GI events. Cohort-restricted reporting odds ratios (RORs) were stratified by calendar quarter. The prespecified acute/postoperative restriction was the most clinically aligned comparison; reporter-occupation restrictions were post hoc. No case-level causality assessment was performed.

Results

The cohort included 894 suzetrigine and 2,365 opioid reports. Respiratory depression appeared in 0/894 and 11/2,365 reports; the primary comparison was non-estimable. Sedation reporting was 33/894 versus 52/2,365 (Mantel-Haenszel ROR 1.77, 95% confidence interval (CI) 1.13-2.76; Holm-adjusted p=0.0244), and GI reporting was 76/894 versus 157/2,365 (ROR 1.31, 95% CI 0.98-1.74; adjusted p=0.0688). The most clinically aligned acute/postoperative restriction attenuated sedation to ROR 1.03 (95% CI 0.56-1.89). In post hoc reporter analyses, consumer-only sedation occurred in 16 of 277 suzetrigine reports and 30 of 1,647 pooled-opioid reports (ROR 3.13, 95% CI 1.66-5.88). Sedation administered exclusively by health care professionals occurred in eight of 295 suzetrigine reports and 17 of 481 pooled-opioid reports, failing to meet the 20-event comparator threshold.

Conclusions

The respiratory comparison was non-estimable. Sedation reporting was elevated in the main cohort; consumer-only estimates were elevated for sedation and GI events. In the acute/postoperative restriction, both domain confidence intervals included 1. These patterns are compatible with reporting bias or channeling but establish neither their cause nor clinical equivalence. Zero respiratory reports do not establish safety. Denominator-based studies are needed.

## Introduction

Suzetrigine (Journavx) is a selective NaV1.8 inhibitor approved by the US Food and Drug Administration (FDA) on January 30, 2025, for adults with moderate-to-severe acute pain [1]. NaV1.8 is expressed in peripheral pain-sensing neurons, so its mechanism differs from that of centrally acting opioids, but mechanism alone cannot predict real-world comparative adverse-event rates. Two phase 3 trials used hydrocodone-acetaminophen and placebo comparators, but trials cannot characterize uncommon or selectively reported events after broader use [2].

Spontaneous-report systems can identify reporting patterns warranting further evaluation but lack exposed-population denominators and cannot establish incidence, relative risk, or causality; reports are also shaped by stimulated reporting, notoriety, channeling, and reporter mix [3].

Broad postmarketing signal-mining studies of suzetrigine already exist, using frequentist and empirical Bayes approaches with non-opioid and background comparators and several disproportionality metrics; earlier opioid-focused studies in the US Food and Drug Administration Adverse Event Reporting System (FAERS) showed variation by molecule, calendar period, reporting channel, and misuse/overdose context [4-8]. These sources of variation matter because pooling different opioids or mixing therapeutic-use and misuse-related reports can change the apparent reporting pattern without demonstrating a difference in clinical risk. Repeating an all-preferred-term scan would add limited novelty.

Active comparators can reduce some distortions of whole-database comparison, although comparator choice can itself introduce competition, masking, and channeling bias [9-12]. In a targeted literature refresh completed July 27, 2026, we found the two direct broad suzetrigine FAERS studies but no concurrent opioid active-comparator analysis of this frozen respiratory/sedation/gastrointestinal (GI) hierarchy. Our primary objective was to compare reporting of the exact MedDRA preferred term Respiratory depression between suzetrigine, a NaV1.8 inhibitor, and pooled oxycodone, hydromorphone, morphine, and tramadol reports. Secondary objectives evaluated the prespecified sedation and GI domains; exploratory analyses evaluated their individual constituent terms and the separate terms Apnea, Hypoventilation, and Respiratory arrest. We hypothesized lower reporting odds for the primary endpoint and secondary domains with suzetrigine. These objectives concern relative reporting among submitted reports, not comparative incidence, clinical risk, or causality.

## Materials and methods

Data source and study period

This was a cross-sectional disproportionality analysis of individual case safety reports. The protocol and analysis plan were frozen before study-outcome inspection on July 24, 2026, and reporting followed the reporting of a disproportionality analysis for drug safety signal detection using individual case safety reports in pharmacovigilance (READUS-PV) [13,14]. We used noncumulative FDA quarterly American Standard Code for Information Interchange (ASCII) extracts from the FAERS/Adverse Event Monitoring System (AEMS) quarterly data portal [15]. The primary window covered 2025 Q2 through 2026 Q1 (April 1, 2025-March 31, 2026); the 2025 Q1 extract was used only for a prespecified partial-launch sensitivity. The exact archived file versions were identified by source URL and SHA-256 checksum; archive integrity and checksums were verified on August 29, 2026. The original download date was not independently recorded. File identifiers and checksums are provided in the reproducibility manifest (Supplementary methods).

The five archives contained 2,014,668 demographic records. Retaining the latest public version per CASEID by greatest FDA receipt date (FDA_DT), followed by greatest numeric PRIMARYID, produced 1,816,428 latest case records and removed 198,240 public versions. Semantic duplicates cannot be fully excluded [16].

Drug mapping and report selection

Drug names and active ingredients were normalized with prespecified, versioned rules. Suzetrigine exposure included suzetrigine, Journavx, and VX-548; the opioid comparator included oxycodone, hydromorphone, morphine, or tramadol. The main cohort required the study drug to be the primary suspect and excluded reports with suzetrigine and an opioid as co-suspects, more than one comparator opioid in the selected suspect role, or hydrocodone as an additional opioid co-suspect (Supplementary methods).

The main cohort was not indication-matched: suzetrigine reports largely reflected acute use, whereas opioid reports could reflect acute or chronic treatment. The comparator comprised four μ-opioid-receptor agonists heterogeneous in mechanism, route, care setting, and dose titration against a fixed suzetrigine dose that FAERS/AEMS does not reliably capture (Supplementary methods). Prespecified tramadol-exclusion and hydrocodone-addition sensitivities addressed part of this mismatch; the remainder is a named limitation.

The primary analysis included US reports, using occurrence country when available and reporter country only when it was missing; an occurrence-country-only sensitivity assessed that choice. Reports documenting an age younger than 18 years were excluded, and reports with missing age were retained to avoid discarding otherwise eligible reports. Their adult status could not be verified, so the cohort is adult or age-missing rather than exclusively adult; differential age missingness could alter the comparison.

To focus the main cohort on therapeutic use, reports containing prespecified indication or reaction terms for abuse, intentional misuse, overdose, poisoning, withdrawal, intentional self-harm, or suicidality were excluded, and a sensitivity analysis restored them. Missing indication was not interpreted as absence of acute pain.

Outcomes

The primary endpoint was the exact FDA-supplied MedDRA preferred-term string Respiratory depression; this narrow, auditable definition avoided changing concept breadth after observing sparse data but may miss clinically related terms. No post hoc respiratory composite or continuity-corrected primary estimate was permitted.

Two prespecified secondary domains were defined as report-level unions. The sedation-related domain comprised reports containing Somnolence, Sedation, or Depressed level of consciousness. The GI domain comprised reports containing Nausea, Vomiting, Constipation, or Ileus.

The prespecified unions summarize reports containing any component term but do not imply that all components have the same direction or magnitude of association. Individual constituent terms and the severe respiratory terms Apnea, Hypoventilation, and Respiratory arrest were evaluated as separate exploratory outcomes. Component-level findings are therefore presented alongside the domain estimates.

We state this event-count gate rule once, explicitly, because it was applied uniformly and without exception to every analysis in this study: the primary endpoint required at least 10 suzetrigine and 20 comparator events, and every secondary domain, exploratory preferred term, prespecified sensitivity analysis, and post hoc reporter-occupation analysis required at least five suzetrigine and 20 comparator events, including descriptive stratum comparisons such as the crude reporter-type-stratum odds ratios in Results. Where a stratum failed this gate, its point estimate is not treated as a reliable indicator of direction or magnitude, even where a numeric value could be computed and is reported for transparency.

The exact MedDRA version is not included in the FDA ASCII files, so no version range was assumed. Exact FDA-supplied preferred-term strings were used without hierarchy remapping, and the event, misuse, indication, and drug mapping tables were frozen before comparative estimation [17].

Statistical analysis

The statistical measures used in this study are defined as follows. The reporting odds ratio (ROR) is a disproportionality measure: the odds that a report for the study drug names the event of interest divided by the same odds among comparator reports. It describes how often an event is reported relative to a comparator within a spontaneous-report database and is not an incidence, a risk, or a risk ratio. The proportional reporting ratio (PRR) is the alternative disproportionality measure, comparing the proportion of reports naming the event between drug and comparator; we did not use it as the primary measure for the reason given below. The Mantel-Haenszel (MH) ROR is a weighted average of the quarter-specific RORs and estimates a common ROR while holding calendar quarter fixed. The Robins-Breslow-Greenland estimator supplies the variance of that weighted average, and the log-Wald interval is the corresponding 95% confidence interval (CI) computed on the logarithmic scale; the Woolf method is the analogous log-scale variance estimator for a single unstratified (crude) ROR. The Breslow-Day test assesses whether the ROR is homogeneous across calendar-quarter strata, and the Woolf test of homogeneity assesses the same across reporter-type strata; both are tests of between-stratum consistency, not of the size of the association. The Holm method is a sequentially rejective correction that controls the family-wise error rate, the probability of at least one false-positive result across a prespecified family of tests. The Benjamini-Hochberg procedure controls the false-discovery rate (FDR), the expected proportion of rejected tests that are false positives. Exact binomial (Clopper-Pearson) calculations were used for one-sided bounds and probabilities involving a single proportion.

For an eligible outcome, we built quarter-specific two-by-two tables and computed the cross-product odds ratio, termed a cohort-restricted ROR to distinguish it from a whole-database case/non-case ROR; the odds-ratio form was preferred over the PRR [18,19]. The primary measure was the MH ROR stratified by calendar quarter, with 95% CIs from the Robins-Breslow-Greenland variance estimator and a log-Wald interval, which is consistent under sparse-data and large-strata limiting models but not thereby guaranteed nominal finite-sample coverage [20-22]. Crude RORs used Woolf log-Wald intervals, and quarter-strata homogeneity was assessed with the Breslow-Day test [23]. Cell definitions and the zero-cell rule are in the Supplementary Statistical details.

The two secondary-domain p-values were adjusted with the Holm method [24] across a family of m=2 prespecified domains. Gate-passing exploratory preferred terms were adjusted with the Benjamini-Hochberg procedure controlling the FDR at 5% under two declared families: a narrower m=4 restricted to gate-passing terms, and a wider m=10 comprising every prespecified exploratory candidate term, with the six gate-failed terms carried as untested and non-rejected [25]. Because defining the family by estimability is anti-conservative in the direction of the reported findings, we take m=10 as primary and report m=4 alongside it (Table 1; Table 2). Information component, empirical Bayes geometric mean, shrinkage-adjusted ROR, and system-organ-class screening were not performed; Table 3 records each gate-based omission and the threshold that triggered it.

**Table 1:** Prespecified secondary and gate-passing exploratory reporting odds analyses, including event counts, MH RORs, nominal p-values, and multiplicity-adjusted p-values Events/total is the number of reports containing at least one qualifying preferred term divided by the total number of reports in that cohort. N refers to individual case safety reports, not patients or events, and each report contributes at most once to a row. m is the number of hypotheses in the relevant multiplicity-adjustment family. Holm-adjusted p-values apply only to the two prespecified secondary domains (m=2). BH-adjusted p-values apply only to exploratory preferred terms: m=4 includes the four terms that passed their event-count gate, whereas m=10 includes all 10 prespecified exploratory candidate terms in the Methods, with the six gate-failed terms carried as untested, non-rejected placeholders. Capped indicates a BH-adjusted p-value truncated at its maximum possible value of 1.00. Under m=10, constipation no longer meets the conventional 5% FDR threshold. MH, Mantel-Haenszel; ROR, reporting odds ratio; CI, confidence interval; BH, Benjamini-Hochberg; n/a, not applicable; FDR, false-discovery rate; GI, gastrointestinal

Analysis	Suzetrigine events/total	Opioid events/total	MH ROR (95% CI)	Nominal p	Adjusted p, Holm m=2	Adjusted p, BH m=4	Adjusted p, BH m=10
Sedation domain (secondary)	33/894	52/2,365	1.77 (1.13-2.76)	0.0122	0.0244	n/a	n/a
GI domain (secondary)	76/894	157/2,365	1.31 (0.98-1.74)	0.0688	0.0688	n/a	n/a
Somnolence (exploratory)	32/894	43/2,365	2.11 (1.32-3.38)	0.00179	n/a	0.00358	0.00895
Nausea (exploratory)	57/894	85/2,365	1.90 (1.34-2.70)	0.000305	n/a	0.00122	0.00305
Vomiting (exploratory)	17/894	55/2,365	0.84 (0.48-1.46)	0.529	n/a	0.529	1.00 capped
Constipation (exploratory)	11/894	55/2,365	0.48 (0.25-0.92)	0.0273	n/a	0.0364	0.0910

**Table 2:** Gate-based omissions This table lists analyses that were prespecified but not estimated, along with the reason for each omission. Where the reason is given as a pair of numbers, these represent event counts for suzetrigine reports and comparator reports, respectively; both fall below the exploratory event-count gate of at least five suzetrigine reports and 20 comparator reports. The primary Respiratory depression endpoint used the separate threshold of at least 10 suzetrigine reports and 20 comparator reports. N refers to individual case safety reports, out of 894 suzetrigine reports and 2,365 pooled-opioid reports. PT, preferred term; ROR, reporting odds ratio; IC, information component; EBGM, empirical Bayes geometric mean

Analysis	Reason
Respiratory-depression ROR	Primary event-count gates failed
Respiratory composite	Prohibited post hoc endpoint substitution
Sedation PT	1 suzetrigine and 9 comparator events
Depressed level of consciousness PT	0 and 2 events
Ileus PT	0 and 0 events
Apnea PT	0 and 0 events
Hypoventilation PT	0 and 2 events
Respiratory arrest PT	0 and 8 events
IC and EBGM	Omitted by protocol; broad signal mining already published
Shrinkage-adjusted ROR	Omitted by protocol
Three-way indication strata	Not performed; the analysis plan specified an acute/postoperative restriction
Weibull time to onset	Not performed because the authorized secondary analysis did not include onset modeling
Covariate-adjusted Firth model	Optional analysis not performed because the complete-case parameter gate was not met

**Table 3:** Gate-passing main analyses: RORs and multiplicity-adjusted p-values N throughout refers to individual case safety reports, not patients or events. The suzetrigine and comparator event columns show the numbers of reports containing at least one qualifying term, out of 894 suzetrigine reports and 2,365 pooled-opioid reports; each report is counted at most once per row. m is the number of hypotheses in the relevant multiplicity-adjustment family. Holm-adjusted p-values apply only to the two prespecified secondary domains (m=2). BH-adjusted p-values apply only to exploratory preferred terms: m=4 includes the four gate-passing terms above, whereas m=10 includes all 10 prespecified exploratory candidate terms, with the six gate-failed terms (Sedation, Depressed level of consciousness, Ileus, Apnea, Hypoventilation, and Respiratory arrest) carried as untested, non-rejected placeholders. Capped indicates a BH-adjusted p-value truncated at its maximum possible value of 1.00. MH, Mantel-Haenszel; ROR, reporting odds ratio; CI, confidence interval; PT, preferred term; BH, Benjamini-Hochberg; n/a, not applicable; GI, gastrointestinal

Family	Outcome	Suzetrigine events	Comparator events	MH ROR	95% CI	Nominal p	Holm-adjusted p (secondary outcomes, m=2)	BH-adjusted p (gate-passing exploratory terms, m=4)	BH-adjusted p (all exploratory terms, m=10)
Secondary	Sedation domain	33	52	1.769	1.132-2.764	0.01221	0.0244	n/a	n/a
Secondary	GI domain	76	157	1.306	0.980-1.740	0.06877	0.0688	n/a	n/a
Exploratory PT	Somnolence	32	43	2.112	1.321-3.377	0.00179	n/a	0.00358	0.00895
Exploratory PT	Nausea	57	85	1.902	1.342-2.696	0.00031	n/a	0.00122	0.00305
Exploratory PT	Vomiting	17	55	0.836	0.480-1.458	0.52859	n/a	0.529	1.00
Exploratory PT	Constipation	11	55	0.479	0.249-0.921	0.02729	n/a	0.0364	0.0910

Prespecified sensitivity analyses evaluated crude versus quarter-stratified estimates; each individual comparator when event gates passed; exclusion of tramadol; addition of hydrocodone; restriction to mapped acute/postoperative-pain indications; exclusion of combination products; inclusion of misuse/overdose-related reports; primary- or secondary-suspect roles; the full 2025 Q1-2026 Q1 period; and occurrence-country-only restriction. The optional covariate-adjusted Firth model and time-to-onset modeling were not run.

Post hoc analyses restricted the main cohort to reports with a known occupation, health care professional (HCP) reports (codes MD, PH, OT, HP), and consumer reports (code CN). These analyses retained the frozen outcome gates and were not added to the Holm family. We also calculated report volumes corresponding to an expected 10 respiratory-depression reports and exact binomial probabilities of observing at least 10 [26]. This scenario is not clinical-event or comparative-ROR power.

No case narrative review or case-level causality assessment was performed. Analyses used Python 3.10 (Python Software Foundation, Wilmington, DE, USA), and all reported estimates were independently recomputed from the exported cell counts (Supplementary methods).

## Results

Cohort construction and feasibility

After latest-version deduplication and the frozen main-cohort rules, 894 suzetrigine and 2,365 pooled-opioid reports were retained (Table 4); the full attrition audit is in Table 5. Mapped acute/postoperative indications occurred in 574/894 (64.2%) suzetrigine versus 437/2,365 (18.5%) opioid reports, confirming that the main cohort was in mixed-indication settings. The opioid pool was 71.7% oxycodone, 14.5% morphine, 7.7% tramadol, and 6.2% hydromorphone.

**Table 4:** Primary cohort size and selected data completeness N refers to individual case safety reports, not patients or events. The column totals are 894 suzetrigine reports and 2,365 pooled-opioid reports. Cells show report counts as n (%), with percentages calculated on the column total. Calendar-quarter rows show counts only and sum to the respective column total. Molecule rows count reports naming that opioid in the selected suspect role within the pooled-opioid column and sum to that column total. Completeness rows count reports in which the listed field was absent. “–” means not applicable because individual comparator molecules are absent from the suzetrigine cohort by design.

Measure	Suzetrigine	Pooled opioids
Total reports	894	2,365
2025 Q2	148	606
2025 Q3	220	697
2025 Q4	244	461
2026 Q1	282	601
Oxycodone	-	1,695 (71.7%)
Morphine	-	343 (14.5%)
Tramadol	-	181 (7.7%)
Hydromorphone	-	146 (6.2%)
Age missing	546 (61.1%)	1,349 (57.0%)
Sex missing	296 (33.1%)	681 (28.8%)
Occurrence country missing	46 (5.1%)	190 (8.0%)
Indication missing	7 (0.8%)	165 (7.0%)
Reporter occupation missing	322 (36.0%)	189 (8.0%)

**Table 5:** Cohort-flow audit Count refers to database records for the first three rows (DEMO-table rows and case versions in the FDA quarterly ASCII extracts) and to individual case safety reports for the final three rows; N never refers to patients or events. The last two rows partition the final primary-window therapeutic-use cohort (894+2,365=3,259 reports). The complete attrition file records every mapping, role, country, age, co-suspect, and misuse-related exclusion. FDA, Food and Drug Administration; ASCII, American Standard Code for Information Interchange

Stage	Count
Raw five-quarter DEMO records	2,014,668
Latest CASEID records retained	1,816,428
Public case versions removed	198,240
Final primary-window therapeutic-use cohort	3,259
Suzetrigine reports	894
Pooled-opioid reports	2,365

Age was missing for 546 of 894 suzetrigine reports (61.1%) and 1,349 of 2,365 opioid reports (57.0%); sex was missing for 296 of 894 suzetrigine reports (33.1%) and 681 of 2,365 opioid reports (28.8%). Reporter occupation was missing for 322 of 894 suzetrigine reports (36.0%) but only 189 of 2,365 opioid reports (8.0%), indicating a material reporting-channel imbalance.

Primary endpoint

The exact MedDRA preferred term Respiratory depression occurred in 0/894 suzetrigine reports and 11/2,365 pooled-opioid reports. Neither primary threshold was met; no ROR, continuity-corrected estimate, or composite measure was calculated. Before the therapeutic use exclusion, which removed eight of the 19 opioid respiratory reports and the sole suzetrigine respiratory report, the counts were 1/902 for suzetrigine and 19/3,558 for pooled opioids; the primary thresholds remained unmet. Zero reports do not establish respiratory safety.

The separate exploratory PT counts were Apnea, 0 suzetrigine versus 0 pooled-opioid reports; Hypoventilation, 0 versus 2; and Respiratory arrest, 0 versus 8 (Table 6). These terms were evaluated separately and were not combined into a post hoc respiratory endpoint. At the observed opioid respiratory-depression proportion, about 2,690 total suzetrigine reports (1,796 additional) would provide an 80% probability of at least 10 reports, and 3,374 total (2,480 additional) a 95% probability. These scenarios quantify report-count feasibility, not clinical-event or comparative-ROR power.

**Table 6:** Respiratory count transparency and feasibility Cells show the number of reports containing the listed preferred term divided by the total number of reports in the relevant arm and cohort restriction. N refers to individual case safety reports, not patients or events. Status describes the prespecified decision for that row: “Primary non-estimable; no ROR” means the primary event-count gate (at least 10 suzetrigine reports and 20 comparator reports) was not met, so no ROR was computed; “Primary still below gates” means the same gate was not met in the wider pre-exclusion cohort; “Separate exploratory PT; no composite” means the term was analyzed only as an individual exploratory preferred term and was not pooled into a respiratory composite. The post hoc feasibility scenarios (4.16 expected events at n=894; approximately 2,150 reports for an expected count of 10; and approximately 2,690 reports for an 80% binomial probability of at least 10 events) are reported in the main text. These scenarios assume that the pooled-opioid report proportion applies to suzetrigine and remains stable. They do not estimate exposed-patient requirements, clinical-event power, or comparative-ROR power. PT, preferred term; ROR, reporting odds ratio

Analysis	Suzetrigine	Pooled opioids	Status
Respiratory depression, main cohort	0/894	11/2,365	Primary non-estimable; no ROR
Respiratory depression (before excluding therapeutic use)	1/902	19/3,558	Primary still below gates
Apnea, main cohort	0/894	0/2,365	Separate exploratory PT; no composite
Hypoventilation, main cohort	0/894	2/2,365	Separate exploratory PT; no composite
Respiratory arrest, main cohort	0/894	8/2,365	Separate exploratory PT; no composite

Prespecified secondary domains

Sedation-domain events occurred in 33/894 (3.7%) suzetrigine and 52/2,365 (2.2%) opioid reports. The quarter-stratified ROR was 1.77 (95% CI 1.13-2.76; nominal p=0.0122; Holm-adjusted p=0.0244), and Breslow-Day did not reject homogeneity across the four calendar quarters (χ²=1.70, 3 df, p=0.637). The domain was driven almost entirely by Somnolence, which accounted for 32 of the 33 suzetrigine and 43 of the 52 domain events; Sedation and Depressed level of consciousness failed their own gates (Table 1), and within-report overlap among the three constituents was negligible.

GI-domain events occurred in 76/894 (8.5%) suzetrigine and 157/2,365 (6.6%) opioid reports. The ROR was 1.31 (95% CI 0.98-1.74; nominal and Holm-adjusted p=0.0688); the CI included 1, and Breslow-Day did not reject homogeneity (χ²=1.95, 3 df, p=0.582). Domain counts are not the sum of the constituent terms in Table 1 because a report could carry more than one, and constituent overlap was greater in the opioid arm, the direction that attenuates rather than inflates the contrast. Unlike sedation, the constituents did not move in a common direction: Nausea was reported more often with suzetrigine (ROR 1.90, 95% CI 1.34-2.70) and Constipation less often (ROR 0.48, 95% CI 0.25-0.92), with non-overlapping intervals.

Exploratory preferred terms and sensitivity analyses

Somnolence, nausea, vomiting, and constipation met the exploratory gates. Somnolence had higher reporting odds with suzetrigine (ROR 2.11, 95% CI 1.32-3.38; BH-adjusted p=0.00895 under the primary m=10 family). GI terms were evaluated separately: nausea occurred in 57/894 suzetrigine versus 85/2,365 opioid reports (ROR 1.90, 95% CI 1.34-2.70; BH-adjusted p=0.00305); vomiting in 17/894 versus 55/2,365 (ROR 0.84, 95% CI 0.48-1.46; BH-adjusted p=1.00); and constipation in 11/894 versus 55/2,365 (ROR 0.48, 95% CI 0.25-0.92; BH-adjusted p=0.0910). These adjusted p-values use the primary m=10 family. The lower constipation estimate is hypothesis-generating because it did not meet the 5% FDR threshold under that family. Ileus, Sedation, Depressed level of consciousness, Apnea, Hypoventilation, and Respiratory arrest failed their gates and were not estimated.

Sedation point estimates exceeded 1 across the gate-passing sensitivities, but the 95% confidence intervals varied in whether they excluded the null value of 1 (Table 7). The intervals excluded 1 when tramadol was excluded, hydrocodone added, combination products excluded, the partial-launch quarter included, or occurrence country defined US reports. They included 1 in the acute/postoperative restriction (ROR 1.03, 95% CI 0.56-1.89), misuse-included cohort, and primary-or-secondary-suspect analysis. An interval including 1 does not establish equivalence or absence of a clinical difference.

**Table 7:** Prespecified secondary-domain sensitivity analyses Event counts are the numbers of suzetrigine and comparator (opioid) reports containing the domain outcome, out of the respective arm totals for each sensitivity-analysis cohort restriction. Arm totals vary across rows because several sensitivities restrict both arms. All counts are summed across the four calendar-quarter strata. Gate failed means that the secondary-domain event-count gate of at least five suzetrigine reports and 20 comparator reports was not met, so no ROR was computed for that cell. The sedation comparator count in the acute/postoperative-indication sensitivity analysis (20/437) just met the comparator event-count gate of 20 events. The gate threshold is 20 comparator events, and the comparator count in this analysis equals that threshold exactly. Sensitivity-analysis p-values were not used to determine the number of confirmations. Direction and interval stability were assessed descriptively. Post hoc reporter-occupation analyses were not adjusted for multiplicity. GI, gastrointestinal; MH, Mantel-Haenszel; ROR, reporting odds ratio; CI, confidence interval; HCP, health care professional

Sensitivity	Sedation suzetrigine events/total	Sedation comparator​​​​​​​ events/total	Sedation MH ROR (95% CI)	GI suzetrigine​​​​​​​ events/total	GI comparator​​​​​​​ events/total	GI MH ROR (95% CI)
Exclude tramadol	33/894	50/2,184	1.70 (1.09-2.67)	76/894	141/2,184	1.33 (0.99-1.78)
Add hydrocodone	33/894	58/2,727	1.87 (1.21-2.90)	76/894	186/2,727	1.28 (0.97-1.69)
Acute/postoperative indication	26/574	20/437	1.03 (0.56-1.89)	54/574	49/437	0.82 (0.54-1.24)
Exclude combination products	33/894	47/2,207	1.85 (1.17-2.92)	76/894	141/2,207	1.36 (1.02-1.82)
Include misuse/overdose	34/902	91/3,558	1.48 (0.99-2.21)	77/902	223/3,558	1.40 (1.06-1.83)
Primary or secondary suspect	33/889	74/2,816	1.44 (0.95-2.18)	74/889	255/2,816	0.91 (0.69-1.19)
Full 2025 Q1-2026 Q1	34/908	65/2,976	1.80 (1.16-2.80)	79/908	201/2,976	1.35 (1.02-1.79)
Occurrence country only	32/848	51/2,175	1.70 (1.08-2.67)	72/848	144/2,175	1.30 (0.97-1.76)
Oxycodone only	33/894	34/1,695	1.96 (1.20-3.20)	76/894	95/1,695	1.57 (1.15-2.16)
Morphine only	33/894	7/343	Gate failed	76/894	34/343	0.84 (0.55-1.29)
Hydromorphone only	33/894	9/146	Gate failed	76/894	12/146	Gate failed
Tramadol only	33/894	2/181	Gate failed	76/894	16/181	Gate failed
HCP reporter only (post hoc)	8/295	17/481	Gate failed	19/295	43/481	0.63 (0.35-1.13)
Known reporter occupation only (post hoc)	24/572	47/2,176	1.94 (1.16-3.22)	49/572	144/2,176	1.31 (0.93-1.85)

Reporter type was strongly unbalanced: among reports with known occupation, 295/572 (51.6%) suzetrigine versus 481/2,176 (22.1%) opioid reports were HCP-sourced. In post hoc consumer-only analyses, sedation was 16/277 versus 30/1,647 (MH ROR 3.13, 95% CI 1.66-5.88) and GI reporting was 30/277 versus 101/1,647 (ROR 1.82, 95% CI 1.18-2.82). In HCP reports, sedation was 8/295 versus 17/481 and failed the comparator gate; GI reporting was 19/295 versus 43/481 (ROR 0.63, 95% CI 0.35-1.13). HCP-consumer heterogeneity was present for sedation (χ²=7.44, p=0.006) and GI reporting (χ²=7.33, p=0.007). Thus, elevations were concentrated in consumer reports, but sparse HCP sedation data do not prove absence among clinicians. These post hoc estimates were not multiplicity-adjusted; full cells are deposited publicly.

GI estimates were heterogeneous: the primary-domain estimate included 1, some alternate cohorts exceeded 1, and the acute/postoperative, primary-or-secondary-suspect, and HCP-only estimates were below or near 1. Among individual comparators, only oxycodone passed the gate for both domains (sedation ROR 1.96, 95% CI 1.20-3.20; GI ROR 1.57, 95% CI 1.15-2.16), directionally consistent with the pooled results; morphine passed for GI only (ROR 0.84, 95% CI 0.55-1.29) and the remaining comparators failed. Quarter-stratified RORs and 95% CIs for both secondary domains and every gate-passing exploratory term are reported in Table 1 and Table 2.

## Discussion

Principal findings

This analysis did not answer the prespecified respiratory-depression question because the required event counts were absent. Zero spontaneous reports cannot justify a favorable safety conclusion, and the feasibility scenarios only estimate the reports needed to clear the count gate. The hypothesis of a broadly lower opioid-type adverse-event profile was not supported.

Sedation-related terms had higher main-cohort reporting odds, whereas the GI confidence included 1. Sedation was driven by Somnolence, while the GI domain combined Nausea moving up with Constipation moving down. Consumer-only RORs were elevated, but HCP sedation failed the event gate, and HCP GI reporting was not elevated; formal heterogeneity indicates strong reporter-channel dependence. This pattern makes a reporting artifact plausible but cannot exclude a genuine tolerability difference or prove absence among clinicians.

Relationship to prior evidence

This analysis differs from the two direct 2026 suzetrigine FAERS studies by using a frozen, concurrent opioid comparator and a narrow event hierarchy rather than broad all-term signal mining or non-opioid/background comparators [4-5]. Because those studies draw on overlapping spontaneous-report data, this analysis does not independently confirm their preferred-term signals.

Opioid pharmacovigilance research has shown differences by molecule, calendar period, misuse context, and reporter channel, and comparator-method studies show that disproportionality changes with the reference group [6-10]. The tramadol-excluded estimate remained similar, so tramadol did not explain the pooled sedation result.

Prespecified sensitivity analyses and a post hoc concomitant-opioid restriction probe different potential biases and do not all point the same way. Excluding suzetrigine reports with a concomitant-opioid drug-record entry left the sedation estimate similar, whereas the acute/postoperative restriction yielded an ROR of 1.03 (95% CI 0.56-1.89); the misuse-included and primary-or-secondary-suspect analyses also had confidence intervals including 1. These findings suggest sensitivity to cohort definition but do not identify or rule out the contributions of indication, suspect-role coding, co-medication, or reporting bias.

FAERS reporting rates are not real-world risks. Sedation and Respiratory depression are mature, labeled opioid effects that may be under-reported as expected, whereas symptoms of a newly launched drug may receive greater attention. Launch-era patterns are not universally described by a simple Weber curve; notoriety can alter disproportionality, and reporter types differ in completeness and vocabulary [27-29]. These mechanisms can change RORs without changing incidence.

Calendar-quarter stratification adjusts for common secular time but cannot make a first-year suzetrigine cohort comparable to decades-old opioids in market maturity, cumulative exposure, awareness, or solicitation. The higher nausea and somnolence reporting odds may reflect this mismatch, differential manufacturer follow-up, or treated-condition mix. Lower constipation reporting is pharmacologically plausible but does not survive adjustment under the wider primary family (Table 2), so it is hypothesis-generating.

Randomized-trial and labeling data offer an external check, though not one directly comparable to a spontaneous-report ROR. In the pivotal phase 3 trials, combined nausea-or-vomiting incidence was lower with suzetrigine than with hydrocodone bitartrate/acetaminophen after abdominoplasty (91 of 448 participants (20.3%) versus 150 of 448 (33.5%)) and bunionectomy (39 of 426 (9.2%) versus 71 of 431 (16.5%)), and the approved label’s most common adverse reactions exceeding placebo include no sedation-domain term [2,30]. This runs opposite to the higher sedation- and nausea-domain reporting odds observed here, a divergence compatible with differential expected-effect reporting, a genuine tolerability difference not captured by the trial population or label threshold, or both.

Clinical and research implications

These findings should not delay clinically indicated use of suzetrigine or substitute for randomized and denominator-based evidence. They identify two priorities: comparative cohorts with prescription or administration denominators to estimate respiratory, sedation, and GI event rates while addressing indication, dose, co-medication, and setting; and continued surveillance using prospectively registered updates.

For clinicians, the practical message is narrow: current FAERS/AEMS data are too sparse to compare respiratory-depression reporting under the frozen design, and the available secondary reporting patterns do not establish a uniformly lower adverse-event burden than that of traditional opioids.

Strengths

Strengths include a protocol frozen before outcome inspection, official noncumulative quarterly files with recorded hashes, latest-version deduplication, a concurrent active comparator, suspect-role and misuse controls, sparse-data gates, quarter stratification, multiplicity adjustment, and independent recomputation. The analysis preserved a failed primary rather than substituting a favorable composite. These controls establish computational fidelity but do not validate the estimand or remove reporting bias.

Limitations

FAERS/AEMS lacks exposure denominators; cohort-restricted RORs measure relative reporting among selected submitted reports, not incidence or relative risk. Reports may be incomplete, unverified, semantically duplicated, selectively stimulated, and affected by notoriety and litigation, and drug use, dose, co-medication, monitoring, and indication are incompletely captured.

Missingness was substantial and differential, particularly for reporter occupation, age, and sex. Reporter channel is the largest unresolved threat: HCP reports comprised 295/572 (51.6%) suzetrigine versus 481/2,176 (22.1%) opioid reports with known occupation, and consumer/HCP estimates diverged for both domains. Because occupation was often missing, stratification cannot fully adjust for this imbalance.

Concomitant opioid use was quantified post hoc but not excluded from the primary cohort: 87 of the 894 suzetrigine reports (9.7%) named a comparator opioid or hydrocodone as a concomitant or secondary-suspect entry, none as primary suspect. A sensitivity analysis restricted to the 807 suzetrigine reports without a recorded comparator-opioid or hydrocodone entry reproduced the sedation finding essentially unchanged (MH ROR 1.72, 95% CI 1.08-2.73, versus 1.77), while the GI estimate attenuated further toward the null (MH ROR 1.17, 95% CI 0.86-1.59, versus 1.31). The converse opioid-coexposed subgroup is not an interpretable contrast and is not reported. The opioid comparator is a pooled construct dominated by oxycodone and morphine (Table 1) in which several individual molecules failed event gates, so it corresponds to no single clinical alternative, and the route, care-setting, and dose heterogeneity described in Methods is not addressed by quarter stratification. Excluding misuse-related terms shrank the cohorts asymmetrically, removing 1,193 of 3,558 opioid reports (33.5%, 3,558 to 2,365 reports) versus eight of 902 suzetrigine reports (0.9%, 902 to 894 reports), which could itself alter reporting odds independent of any true safety difference.

Some gate-passing analyses had event counts only modestly above the prespecified minimums, and asymptotic MH confidence intervals can be anti-conservative in sparse strata, so estimates near the gates, including several sensitivity and all reporter-stratified analyses, should be read as indicative rather than precise.

Exposure-duration channeling is unresolved: suzetrigine is used for short-course acute pain, whereas the opioid pool includes acute and chronic treatment. Dose, dosing frequency, treatment duration, and titration were not standardized or adjusted for in this analysis and are incompletely captured in FAERS/AEMS. Consequently, differences in exposure intensity or duration could contribute to the observed reporting patterns. The acute/postoperative restriction was the most clinically aligned comparison and moved sedation to an ROR of 1.03, but its 95% CI (0.56-1.89) does not prove no difference, and indication fields do not establish duration.

The exact MedDRA version was unavailable in ASCII, and no hierarchy remapping was performed. The exact-PT respiratory endpoint may omit clinically related concepts; broadening it after inspection would create a different, post hoc endpoint. Mapping rules require clinician-author confirmation, and combination recognition cannot recover every unstructured variant. The 2026 direct FAERS publications limit novelty and use overlapping data; this is an incremental active-comparator analysis, not a first safety profile.

## Conclusions

The respiratory comparison was non-estimable. Sedation reporting was higher in the main cohort, whereas the GI domain confidence interval included 1. Consumer-only estimates were elevated for both domains, and both acute/postoperative confidence intervals included 1. These patterns are compatible with channeling or reporting bias but do not establish their cause, clinical equivalence, or comparative safety. Individual GI terms showed different reporting patterns. These findings are hypothesis-generating; zero respiratory reports do not establish safety. Confirmation requires longitudinal studies with exposure denominators and adjustment for relevant confounders.

## Appendices

Supplementary Methods

Frozen Hierarchy and Gate Response

The exact preferred term, Respiratory depression, was the prespecified primary endpoint. It occurred in 0/894 suzetrigine reports and 11/2,365 pooled-opioid reports after therapeutic use exclusions, below the required thresholds of 10 and 20 events, respectively. No primary reporting odds ratio (ROR) was calculated. Before these exclusions, the counts were 1 and 19, respectively, and still failed the gate.

The sedation and gastrointestinal unions were prespecified secondary domains. After the primary gate failed, a separate validation step authorized the planned analyses of these domains, gate-passing exploratory preferred terms, and prespecified sensitivities. Respiratory composites, continuity-corrected protective estimates, information component, empirical Bayes geometric mean, shrinkage ROR, system-organ-class screening, three-way indication strata, and time-to-onset modeling remained prohibited.

Exact Outcome Definitions

•       Respiratory primary: Respiratory depression.

•       Sedation domain: Somnolence, Sedation, or Depressed level of consciousness.

•       Gastrointestinal domain: Nausea, Vomiting, Constipation, or Ileus.

•       Severe respiratory exploratory preferred terms: Apnoea, Hypoventilation, or Respiratory arrest.

Each latest-version report was counted once per exact preferred term and once per domain union.

Reproducibility Details

The analysis used noncumulative Food and Drug Administration (FDA) quarterly American Standard Code for Information Interchange (ASCII) archives for 2025 Q2 through 2026 Q1, with 2025 Q1 used only for the partial-launch sensitivity. The archived filenames are faers_ascii_2025q2.zip, faers_ascii_2025q3.zip, faers_ascii_2025Q4.zip, faers_ascii_2026q1.zip, and faers_ascii_2025q1.zip. File identity is fixed by the FDA source URLs and SHA-256 checksums in the reproducibility manifest. Archive integrity and checksums were verified on August 29, 2026; the original download date was not independently recorded.

The complete mapping dictionaries are supplied as outputs/mappings/drug_name_map.csv, event_term_map.csv, misuse_term_map.csv, acute_indication_map.csv, and reporter_occupation_codebook.csv in the reproducibility materials identified under Public reproducibility materials. These files, together with the analysis code, specify the drug-name and ingredient mappings, exact event terms, misuse exclusions, indication restriction, and reporter classification. The main analysis used reports in which the study drug was the primary suspect; concomitant entries alone did not define exposure. The stated co-suspect and multiple-opioid exclusions were applied before analysis. Deduplication retained the greatest FDA_DT, then the greatest numeric PRIMARYID, within each CASEID across the five quarterly files. Reports with missing age were retained, whereas reports with a documented age below 18 years were excluded. US occurrence country was used when available; otherwise, reporter country was used. These rules define a report cohort and do not establish individual exposure histories.

Statistical Details

For quarter q, a_q denotes suzetrigine reports containing the outcome, b_q suzetrigine reports without it, c_q comparator reports containing it, and d_q comparator reports without it; n_q=a_q+b_q+c_q+d_q. The crude ROR is ad/(bc), using summed cells. The Mantel-Haenszel estimate is ROR_MH=R/S, where R=sum(a_q*d_q/n_q) and S=sum(b_q*c_q/n_q).

The Robins-Breslow-Greenland variance of log(ROR_MH) is V=0.5*[sum(U_q)/R^2 + sum(V_q)/(R*S) + sum(W_q)/S^2], where U_q=(a_q+d_q)*a_q*d_q/n_q^2, V_q=[(a_q+d_q)*b_q*c_q+(b_q+c_q)*a_q*d_q]/n_q^2, and W_q=(b_q+c_q)*b_q*c_q/n_q^2. The 95% CI is exp[log(ROR_MH) +/- 1.959964*sqrt(V)]. Two-sided nominal p-values use the standard normal statistic log(ROR_MH)/sqrt(V). Crude log-Wald intervals use variance 1/a+1/b+1/c+1/d when every summed cell is positive.

The primary Respiratory depression endpoint required at least 10 suzetrigine and 20 comparator event reports; secondary domains, exploratory terms, sensitivities, and post hoc reporter analyses required at least five and 20, respectively. Gate-failed analyses were not assigned inferential ROR estimates. No continuity correction was used. Nonempty quarter strata retained their literal zero cells in the Mantel-Haenszel sums. If R or S was zero, or the estimated log variance was nonpositive, the implemented estimator returned no estimate or CI; crude estimates were withheld if any summed cell was zero. A zero event count was not interpreted as evidence of safety.

Holm adjustment used the two prespecified secondary domains. Exploratory Benjamini-Hochberg adjustment is reported for the four gate-passing terms and, as the primary exploratory family, all 10 candidate terms, with the six gate-failed terms retained as untested, non-rejected placeholders. The wider-family calculation uses p=1 placeholders for those six terms solely for multiplicity bookkeeping. For ordered p-values, adjusted p_(i)=min[1, min over j>=i of (m*p_(j)/j)]. Reporter analyses were post hoc and not multiplicity-adjusted. Calendar-quarter stratification does not adjust for indication, exposure duration, dose, or differential reporting. Quarter-specific cells and gate decisions are supplied in the archived aggregate outputs.

Public Reproducibility Materials

The single-file analysis code, frozen protocol, sanitized FDA archive checksums, and aggregate reproducibility outputs (cohort attrition, gate metrics, mapping dictionaries, and the exact cell counts underlying every reported estimate) are permanently archived at https://doi.org/10.5281/zenodo.22164674 (release v1.1.0), with the corresponding source repository at https://github.com/Tigerpoint/suzetrigine-faers-opioid-analysis (commit 7965a2026d2ac075290153b4ecd8cf70433ff5f1). Raw FDA quarterly archives are not redistributed; official source URLs and SHA-256 checksums are provided. No patient-level exports or licensed MedDRA hierarchy is included. 
